# Identification, Isolation, and Characterization of a New Degradation Impurity in Nafcillin Sodium

**DOI:** 10.3797/scipharm.1408-03

**Published:** 2014-10-15

**Authors:** Jagadeesh Kumar Vundavilli, Pavan Kumar S. R. Kothapalli, Badarinadh Gupta Peruri, Prasada Rao V. V. Korrapati, Hemant Kumar Sharma, Sreenivas Nallapati

**Affiliations:** Aurobindo Pharma Limited Research Centre-II, Survey No: 71 & 72, Indrakaran village, Sangareddy mandal, Medak district, 502329, Telangana, India

**Keywords:** Nafcillin Sodium, Stability, Impurities, Characterization

## Abstract

A new degradant of Nafcillin Sodium was found at a level of 1.8% w/w during the gradient reversed-phase HPLC analysis in stability storage samples. This impurity was identified by LC-MS and was characterized by ^1^H-NMR, ^13^C-NMR, LC/MS/MS, elemental analysis, and IR techniques. Based on the structural elucidation data, this impurity was named as *N*-[(2*S*)-2-carboxy-2-{[(2-ethoxynaphthalen-1-yl)carbonyl]amino}ethylidene]-3-({*N*-[(2-ethoxynaphthalen-1-yl)carbonyl]glycyl}sulfanyl)-D-valine. This impurity was prepared by isolation and was co-injected into the HPLC system to confirm the retention time. To the best of our knowledge, this impurity has not been reported elsewhere. The identification and structural elucidation of this degradant impurity has been discussed in detail.

## Introduction

**Fig. 1:**
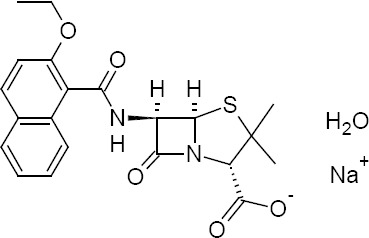
Chemical structure of Nafcillin Sodium

Nafcillin Sodium, chemically known as sodium (2*S*,5*R*,6*R*)-6-{[(2-ethoxynaphthalen-1-yl)carbonyl]amino}-3,3-dimethyl-7-oxo-4-thia-1-azabicyclo[3.2.0]heptane-2-carboxylate hydrate, is a narrow-spectrum beta-lactam antibiotic [1] of the penicillin class and it is derived from 6-Aminopenicillanic acid [2–4]. It is administrated orally and by intramuscular and intravenous injections. As a beta-lactamase-resistant category penicillin, it is used to treat infections caused by Gram-positive bacteria, in particular, species of staphylococci that are resistant to other penicillins. The chemical structure of Nafcillin Sodium is shown in Fig. 1.

It is considered therapeutically equivalent to the Oxacillin Sodium drug, although its safety profile is somewhat different [5]. United States clinical practice guidelines recommend either Nafcillin or Oxacillin as the “first line” treatment of choice for staphylococcal endocarditis in patients without artificial heart valves. It is available as an injection under the trade names Nallpen and Unipen [6].

A number of synthetic procedures in journal articles and patents were reported in the literature for the preparation of Nafcillin and its sodium salt. Chinese scientist Zhang Weiping *et al*. worked on the preparation of Nafcillin Sodium and they were published in Chinese articles during the period between 2009 and 2010 [7–9]. In 2006, Korrapati Prasada Rao *et al*. reported the synthesis of Nafcillin Sodium and its related substances which were obtained during the synthetic process [10].

Several analytical methods have been reported in the literature for the determination of Nafcillin and/or its related substances [11–13]. The USP has also published the monographs for both active pharmaceutical ingredients and formulation products and has given the methodologies for determining the assay of Nafcillin [14]. A stability study of Nafcillin Sodium was evaluated in the literature [15–17]. Studies in the presence of lidocaine hydrochloride reveal that admixtures of Nafcillin 20 mg/mL and lignocaine hydrochloride 0.6 mg/mL in either dextrose 5% or sodium chloride 0.9% are stable for 48 hours at 22–23°C [16].

The formation of degradation impurities during stability storage has been studied and presented in this article. In this study, Nafcillin Sodium was subjected to stress and formal stability storage conditions as per ICH [18, 19]. During the course of this research work, we have analyzed Nafcillin Sodium stability samples; one unknown impurity was observed along with the known impurities at the relative retention time of around 1.26 with respect to the Nafcillin peak [10]. The present research work explains the identification, isolation, structural elucidation, and formation of this degradation impurity.

## Experimental

### Chemicals, Reagents, and Samples

The Nafcillin Sodium drug substance and other known related substances (for the confirmation of retention times in HPLC) were gifted from the Aurobindo Pharma Research Centre. Potassium dihydrogen orthophosphate, acetonitrile, orthophosphoric acid, hydrochloric acid, sodium hydroxide, trifluoroacetic acid, and potassium bromide (IR spectroscopy grade) were purchased from Merck Limited, and pure Milli-Q water was used with the help of the Millipore purification system.

### High-Performance Liquid Chromatography

#### Preparation of Solutions

Dissolve 1.36 g of potassium dihydrogen orthophosphate in 1 litre of water (10 mM) and adjust the pH to 3.5(±0.05) units with dilute orthophosphoric acid (5 mL of orthophosphoric acid in 100 mL of water). Filter the solution through a 0.45 µm porosity membrane filter to be used as mobile phase A. Mobile phase B is acetonitrile and the diluent is water.

#### Sample Solution

Accurately weigh and transfer about 50 mg of the Nafcillin Sodium sample into a 50-mL clean, dry volumetric flask, add 30 mL of diluent, sonicate to dissolve, and dilute to volume with the diluent. Filter the solution through a 0.45 µm porosity membrane filter.

#### Chromatographic Conditions

Column: YMC ODS-AQ, 150 mm long, 4.6 mm i.d., with 5 µm particle size; injection volume: 10 µl; flow rate: 1.2 mL/min; column oven temperature: 30°C; UV detection: 225 nm; data acquisition time: 50 min; pump is in gradient mode and the program is as follows:

time (min)/ A (v/v): B(v/v); T_0.01_/75:25, T_40_/35:65, T_50_/35:65,T_51_/75:25,T_60_/75:25.

### LC/MS/MS Analysis

#### Preparation of Solutions

Mix 1 mL of trifluoroacetic acid in 1000 mL of water. Filter the solution through a 0.45 μm porosity membrane filter to be used as mobile phase A and mobile phase B is acetonitrile.

#### Sample Solution

Accurately weigh and transfer about 100 mg of the Nafcillin Sodium sample into a 50-mL clean, dry volumetric flask, add 30 mL of water, sonicate to dissolve, and dilute to volume with water. Filter the solution through a 0.45 µm porosity membrane filter.

#### LCMS Conditions

TurboIonSpray voltage: 5.5 kV; temperature: 375°C; auxiliary gas and curtain gas: highly pure nitrogen; nebulizer gas: zero air; dwell time: 2.0 s; LC-MS spectra acquired: *m/z* 100–1000 in 0.1 amu steps; column: YMC PACK ODS-A, 150 mm long, 4.6 mm i.d., 5 µm particle size; UV detection: 225 nm; flow rate: 1.0 mL/min; data acquisition time: 40 min; pump is in gradient mode and the program is as follows:

time (min)/ A (v/v): B(v/v); T_0.01_/75:25, T_40_/35:65, T_50_/35:65,T_51_/75:25,T_60_/75:25.

### Preparative Liquid Chromatography

#### Preparation of Solutions

Prepare 1% aqueous ammonium acetate solution (130 mM). Filter the solution through a 0.45 µm porosity membrane filter to be used as mobile phase A and mobile phase B is acetonitrile.

#### Methodology

The column for the isolation of the required impurity: Hyperprep HS C_18_ 250 mm long, 21.2 mm i.d., (make: Thermo Scientific) packed with 8 µm particle size; flow rate: 30 mL/min; UV detection: 225 nm; pump is in gradient mode and the program is as follows:

time (min)/ A (v/v): B(v/v); T_0.01_/100:0, T_20_/95:5, T_40_/90:10,T_60_/85:15, T_80_/82:18, T_100_/100:0, T_120_/20:80.

### Nuclear Magnetic Resonance (NMR)

The solvent used for the experiments: dimethyl sulfoxide (DMSO-d_6_); internal standard: tetramethyl silane (TMS). For the ^1^H-NMR & ^13^C-NMR spectrometers’ operating frequencies: 300.1315 MHz and 75.4748 MHz; number of scans: 32 and 1,062, respectively. SI 32768, LB 0.3 Hz, and SF 300 MHz are the parameters used for data processing.

### Fourier Transform Infrared Spectroscopy (FTIR)

Triturate about 3 mg of the sample in about 400 mg of potassium bromide (previously dried at 80°C). Prepare the pellet using a pellet maker by applying 8-10 tons of pressure and record the IR spectrum between 650 and 4000 cm^−1^ by doing the blank correction by using the KBr pellet.

### Stress Stability Studies

As stated in the ICH guidelines, stability testing is to be carried out to identify the likely degradation products or to elucidate the stability characteristics of the drug substance.^18^ Thermal exposure studies were carried out on Nafcillin Sodium. These experiments and results are discussed in the next sections.

#### Thermal

Nafcillin Sodium samples were subjected to dry heat thermal exposure at 105°C for 150 hrs. Another set of samples were subjected to thermal exposure at 80°C and 60°C on different time intervals *viz*., 15 days and 1 month, respectively.

#### Hydrolytic Condition

The sample solution was mixed with 5 mL of 1 M hydrochloric acid solution, considered as an acidic hydrolysis condition (1 M HCl/ initial / at ambient temperature). The sample solution was mixed with 5 mL of 1 M sodium hydroxide solution, considered as an alkaline (base) hydrolysis condition (1 M NaOH/ initial / at ambient temperature).

#### Oxidative Condition

The sample solution was mixed with 5 mL of 30% hydrogen peroxide (H_2_O_2_) solution and kept at room temperature for 10 min.

#### Photolytic Condition

Nafcillin Sodium samples were exposed to photolytic degradation i.e., 10 K Lux for 150 hours.

#### Humidity Condition

Nafcillin Sodium samples were exposed to 92% relative humidity at 25°C for 150 hours.

### Formal Stability Studies

Nafcillin Sodium samples were also subjected to conditions to evaluate the degradation under formal temperature and humidity conditions, i.e. 40°C(±2°) / 75% RH(±5%) and 25°C(±2°) / 60% RH(±5%), and a range of time intervals (from 1 to 6 months at accelerated storage, and 3 to 12 months at long-term storage). All these samples were packed in aluminum bottles of 50 mL capacity, closed with a chlorobutyl rubber stopper, and finally sealed with an aluminum tear off cap. All samples were withdrawn periodically from their respective environmental conditions. The sample solutions were prepared and injected into the HPLC using the procedure mentioned in the HPLC section.

## Results and Discussion

### Detection and Identification of the Impurity

The sample solutions described under the stress stability conditions were diluted to the necessary concentration and injected into the HPLC using the analytical conditions. From the thermal and humidity chromatograms, an unidentified impurity was found along with known impurities^10^ at a relative retention time of around 1.26 min with respect to the Nafcillin peak, and also the same impurity was observed in the accelerated stability storage conditions. Remarkably, identical results of the impurity levels were observed at around the 1.8% w/w and 2.5% w/w levels in the thermal degradation condition & at around the 0.7% w/w and 0.4% w/w levels in the humidity degradation condition in Nafcillin Sodium.

In formal degradation conditions, this degradation impurity was observed at around the 0.05% w/w to 0.16% w/w level in Nafcillin Sodium in different time intervals.

**Fig. 2:**
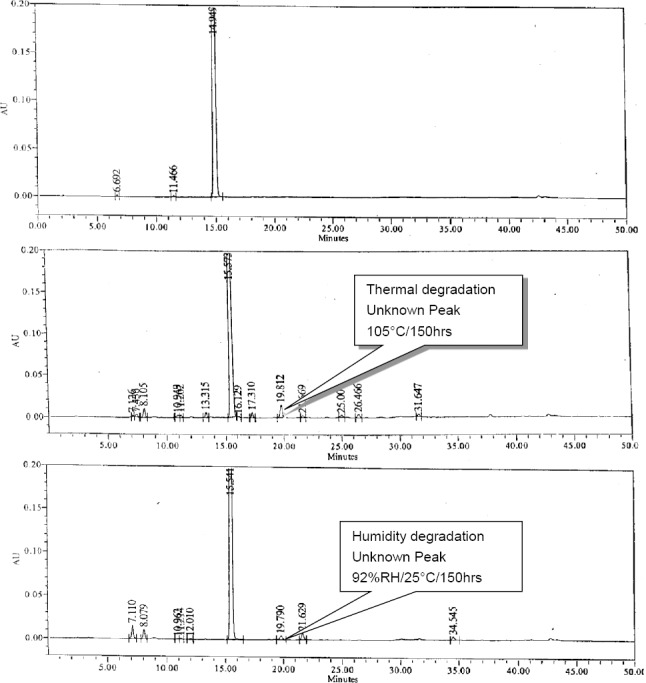
Typical HPLC chromatograms of the Nafcillin Sodium control sample along with the thermal and humidity degradation samples

The degradation impurity formed under the storage conditions is shown in the typical HPLC chromatograms in Fig. 2. The unknown impurity at the relative retention time of around 1.26 min shows the *m/z* value of 688 when subjected to LC/MS analysis. This unknown impurity was collected in a little amount by the preparative isolation method and co-injected into the HPLC system to confirm the relative retention time. Further, this impurity was isolated by preparative liquid chromatography. The preparation and isolation of the impurity is elucidated in the following sections.

### Isolation of the Impurity

The electrospray ionization mass spectrum of the unknown impurity shows the *m/z* value 688 [(M+H)^+^] in positive ion mode by LC/MS analysis. To prepare this impurity in Nafcillin Sodium, the sample was exposed to degrade under 105°C for 150 hours and analyzed by HPLC, the required impurity was observed at around 1.8% w/w. This degraded Nafcillin Sodium sample (about 800 mg) was taken in a beaker and about 10 mL of a phosphate buffer of pH 7.5 (by dissolving 1.74 g dipotassium hydrogen phosphate in 800:200:200 v/v/v water, methanol, and acetonitrile solution) was added to dissolve it. This solution was loaded into the preparative column using the conditions mentioned in the “Preparative Liquid Chromatography” section. Fractions of the impurity ≥90% (time window at around 18–22 min) were pooled mutually, and concentrated on the Rotavapor to remove the organic solvent. The concentrated fractions were loaded into a preparative column and eluent was treated with water to remove the buffer used in the isolation. Finally, the column was washed with water and acetonitrile in the ratio of 50:50 v/v. Again, the eluent was concentrated using the Rotavapor to eliminate the acetonitrile. The aqueous solution was lyophilized using the Virtis advantage 2XL freeze dryer. The impurity was achieved with chromatographic purity of about 80.0% determined by the HPLC method.

### Structural Elucidation of the Impurity

**Fig. 3:**
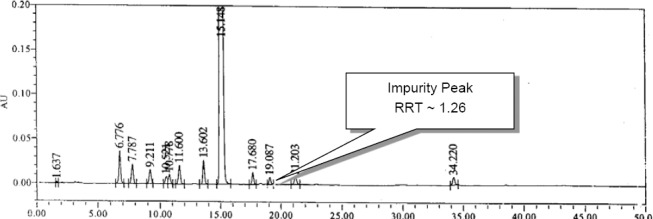
A typical HPLC chromatogram of Nafcillin Sodium spiked with known impurities along with the new impurity

This degradation impurity was co-injected with Nafcillin Sodium into the HPLC to confirm the retention time and relative retention time. The typical HPLC chromatogram of Nafcillin Sodium spiked with known impurities along with the new impurity is shown in Fig. 3.

**Fig. 4:**
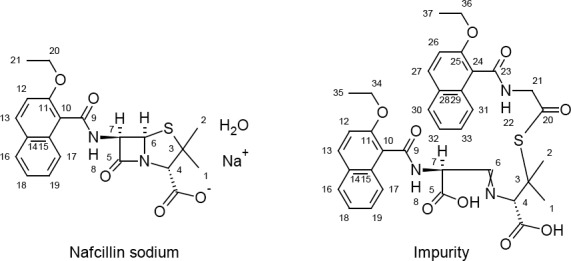
Chemical structure of Nafcillin sodium and the new impurity

The ESI mass spectra of the impurity demonstrates the protonated molecular ion at *m/z* 688.1 amu [(M+H)^+^] in positive ion mode; the Nafcillin protonated molecular ion at *m/z* 413.2 amu [(M-H)^−^] in negative mode, the difference of these two mass values is around 275 amu units. The chemical atom positions of Nafcillin Sodium and the proposed structure of the impurity are represented in Fig. 4. The chemical shift (δ) values [the comparative NMR data is presented in Table 1] obtained from ^1^H-NMR and PENDANT NMR of Nafcillin and the impurity infers the following observations.

**Tab. 1:**
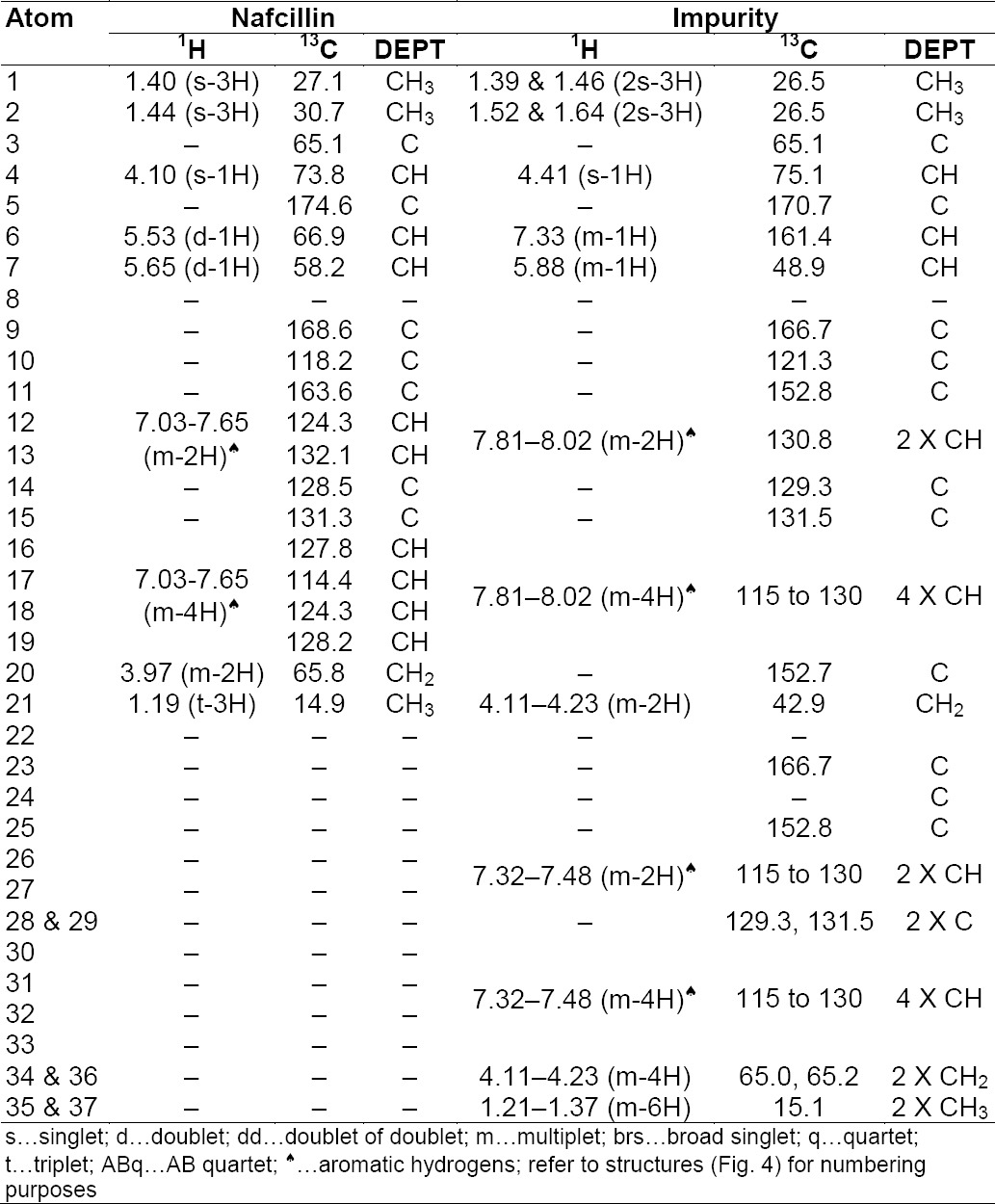
^1^H-, ^13^C-NMR assignments for Nafcillin and the impurity

In the impurity spectra, multiplet signal values were observed at 7.33 ppm and 161.4 ppm which corresponds to ethylene =CH at position 6, while in Nafcillin, 5.53 ppm and 66.9 ppm corresponds to the methyne –CH signal at the same position. Additionally, substantial downfield chemical shifts were observed at position 7, from 5.53 ppm to 5.88 ppm in ^1^H-NMR, and shifts from 58.2 ppm to 48.9 ppm in ^13^C-NMR gets the information about ring opening in the impurity structure.

In the impurity spectra at position 21, the ^1^H-NMR and ^13^C–NMR (PENDANT) chemical shift values at 4.11 ppm, 4.23 ppm, and 42.9 ppm correspond to the methylene -CH_2_ signal as well as at position 20 in the impurity, the absence of the ^1^H-NMR signal and 42.9 ppm in ^13^C-NMR was observed. This depicts that the impurity may have both the penilloic and penicilloic of the Nafcillin moieties.

Further, the impurity structure having two aromatic regions and 12 hydrogen atoms existed in the benzonoid system, which was confirmed through the ^1^H-NMR and ^13^C-NMR signals due to the 12, 13, 16–19, 26, 27, and 30–33 positions, whereas in Nafcillin, only six aromatic hydrogens are present. This phenomenon explains that the dimerization of Nafcillin may take place during degradation.

**Fig. 5:**
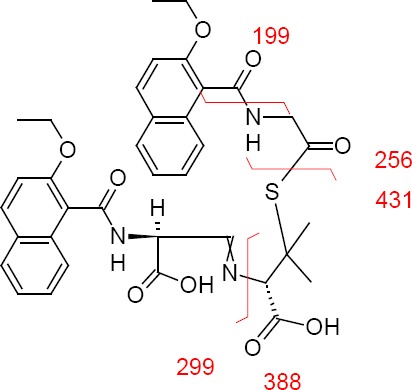
Fragmentation outline of the impurity

Based on the elemental analysis, the theoretical values: C: 62.94; H: 5.43; N: 6.12; S: 4.67 and established values: C: 62.87; H: 5.39; N: 6.08; S: 4.65 recommended that the elemental composition is C_36_H_37_N_3_O_9_S with a molecular weight of 687 and is supported by the attendance of major fragments at m/z 199, 256, 389, and 433, obtained from the mass spectra. The fragmentation is shown in Fig. 5.

In addition to that, this impurity was further confirmed by FTIR spectral data. Comparative FTIR data of the impurity with Nafcillin are summarized in Table 2 and the IR spectra of Nafcillin and the impurity are given below. Based on these spectral data, the impurity is chemically named as *N*-[(2*S*)-2-carboxy-2-{[(2-ethoxynaphthalen-1-yl)carbonyl]amino}ethylidene]-3-({*N*-[(2-ethoxynaphthalen-1-yl)carbonyl]glycyl}sulfanyl)-D-valine.

**Tab. 2:**
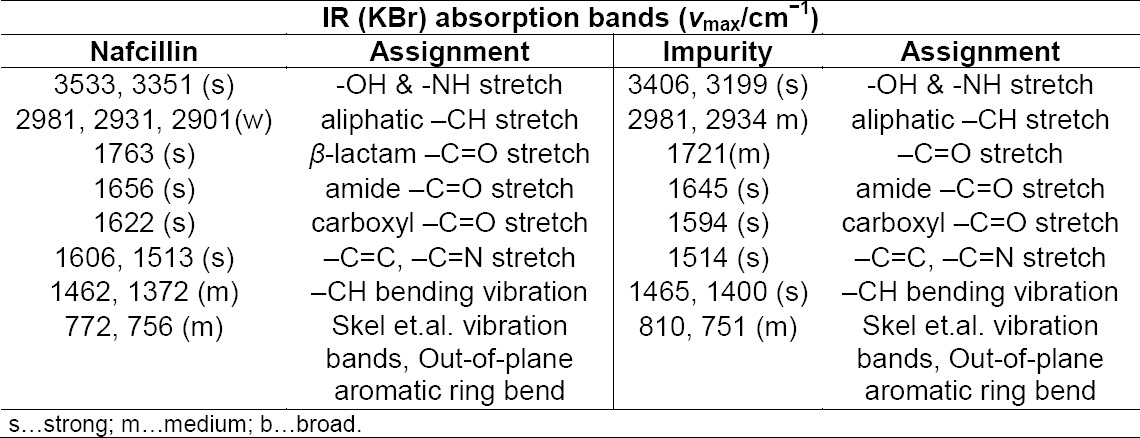
FTIR spectral data of Nafcillin and the impurity

### Formation of the Impurity

**Fig. 6:**
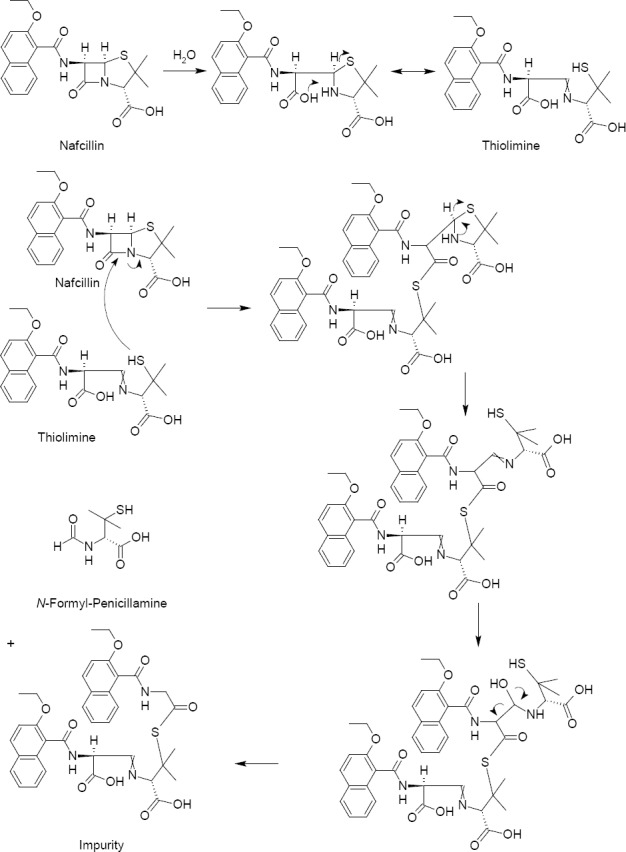
Mechanism for the formation of the impurity

This degradation product of Nafcillin originates from the hydrolytic opening of the β-lactam ring in Nafcillin and is followed by the rearrangement of the penicillin moiety, and thereafter the intramolecular cleavage of β-lactam of Nafcillin by the thiol group, as shown in the mechanism in Fig. 6.

## Conclusion

The Nafcillin Sodium drug substance samples were subjected to stress stability studies and the degradation product was evaluated. One unknown impurity was identified, isolated, and characterized by various spectroscopic techniques like FTIR, NMR, and MS. The most probable structure is proposed for the impurity based on the available spectral data.
